# Influence of Mo Segregation at Grain Boundaries on the High Temperature Creep Behavior of Ni-Mo Alloys: An Atomistic Study

**DOI:** 10.3390/ma14226966

**Published:** 2021-11-18

**Authors:** Qian Li, Jiayong Zhang, Huayuan Tang, Hongwu Zhang, Hongfei Ye, Yonggang Zheng

**Affiliations:** 1 School of Science, North University of China, Taiyuan 030051, China; qian.li@nuc.edu.cn; 2 International Research Center for Computational Mechanics, State Key Laboratory of Structural Analysis for Industrial Equipment, Department of Engineering Mechanics, Faculty of Vehicle Engineering and Mechanics, Dalian University of Technology, Dalian 116024, China; zhangjiayong@mail.dlut.edu.cn (J.Z.); zhanghw@dlut.edu.cn (H.Z.); yehf@dlut.edu.cn (H.Y.); 3 Department of Physics and Astronomy, Clemson University, Clemson, SC 29634, USA; huayunt@clemson.edu

**Keywords:** creep behavior, segregation, strain rate, molecular dynamics simulation, Ni-Mo system

## Abstract

Based on molecular dynamics simulations, the creep behaviors of nanocrystalline Ni before and after the segregation of Mo atoms at grain boundaries are comparatively investigated with the influences of external stress, grain size, temperature, and the concentration of Mo atoms taken into consideration. The results show that the creep strain rate of nanocrystalline Ni decreases significantly after the segregation of Mo atoms at grain boundaries due to the increase of the activation energy. The creep mechanisms corresponding to low, medium, and high stress states are respectively diffusion, grain boundary slip and dislocation activities based on the analysis of stress exponent and grain size exponent for both pure Ni and segregated Ni-Mo samples. Importantly, the influence of external stress and grain size on the creep strain rate of segregated Ni-Mo samples agrees well with the classical Bird-Dorn-Mukherjee model. The results also show that segregation has little effect on the creep process dominated by lattice diffusion. However, it can effectively reduce the strain rate of the creep deformation dominated by grain boundary behaviors and dislocation activities, where the creep rate decreases when increasing the concentration of Mo atoms at grain boundaries within a certain range.

## 1. Introduction

In the past few decades, nanocrystalline (NC) materials have attracted more and more attention because of their excellent properties such as ultra-high strength at room temperature [1,2,3,4,5,6,7]. However, when functioned at high temperatures and under continuous stresses, creep deformation, which may cause accidental deformation or even failure of materials and structures [8,9,10], is an inevitable important issue. Thus, extensive researches have been performed to study the creep mechanism of nanometallic materials, through both experiments and simulations [9,10,11,12,13,14,15,16,17,18].

Previous simulation results have clarified that the main creep mechanisms in NC materials are the lattice diffusion, grain boundary (GB) diffusion, GB sliding and dislocation activity. Different applied stresses may activate different creep mechanisms [9,19,20]. The well-known Brid-Dorn-Mukherjee classical equation [21], which comprehensively describes the effects of external stress, temperature and grain size, is widely used to analyze the creep behavior of NC materials. Its expression is as follows [21]:

(1) $$\dot{\varepsilon }=\frac{AD_{0}Gb}{k_{B}T}{\left(\frac{b}{d}\right)}^{p}{\left(\frac{\sigma }{G}\right)}^{n}\exp\left(-\frac{\Delta Q}{k_{B}T}\right)$$

where $\dot{\varepsilon }$ is the creep strain rate of the material in the steady-state creep stage, A is a dimensionless constant, $D_{0}$ is the diffusion coefficient, G is the shear modulus of the material, b is the magnitude of the Burgers vector, *k_B_* is the Boltzmann constant, T is the absolute temperature, d is the average grain size of the polycrystalline material, σ is the applied stress, ΔQ is the activation energy of a specific thermally activated creep mechanism, and p and n are the grain size exponent and stress exponent, respectively. Among them, the exponents n and p play an important role in determining the creep mechanism of materials. Specifically, different exponents correspond to different creep mechanisms. When *n* = 1, the creep deformation is always dominated by diffusion, and in this case, if *p* = 2, the vacancy diffusion occurs in the crystal, which is usually called the lattice diffusion (i.e., the Nabarro-Herring diffusion) [22]; if *p* is equal to 3, the vacancies flow along the GBs, which is called the GB diffusion, that is, the Coble diffusion [13]. When the stress exponent *n* = 2 or the grain size exponent *p* = 3, the creep process is dominated by the GB slip [12,19]. At the same time, it is worth noting that, the diffusion and slip of GBs always occurs simultaneously [19,23]. When the stress exponent n is larger than 4, the nucleation, slip, and climb of dislocations are the dominant creep deformation mechanisms [23], which is called as the dislocation creep.

One of the most intriguing properties of NC materials that are utilized at various applications is the ultra-high strength [24,25]. It is well accepted that its ultra-high strength is due to the existence of a large number of GBs and the related structures in the crystal. Generally speaking, the strength of NC materials increases with the decrease of grain size. However, when the grain size is refined to a certain extent, for example, about 10 nm to 30 nm, NC materials will soften, i.e., the strength will decrease with the further decrease of grain size [26]. This is mainly caused by the transition of plastic deformation mechanism from dislocation activities to GB behaviors [27]. At the same time, due to the large number of GB structures in NC materials, these materials are sensitive to high temperatures. The instability of GBs also greatly limits the application of NC materials in high temperature areas [28].

Accordingly, strategies against the softening behavior of NC materials caused by GB instability at extremely fine grain size are essential for the potential applications, which have attracted continuous efforts. The experimental results of Lu et al. showed that adding the second-phase atoms and inducing them to segregate towards GBs can effectively avoid the softening phenomenon [29]. Molecular dynamics simulations performed by Sellers et al. also showed that the segregation of Cu and Ag atoms at the GBs of β-Sn material affects the GB energy and shear stress [30]. The study conducted by Meyers et al. suggested that the segregated impurity atoms can pin the GBs, thus reducing the mobility of the GBs [31]. Based on these studies, it can be known that the segregation of the second-phase atoms to GBs has a significant effect on the GB behaviors. That is, the GB segregation effect can change the mechanical properties of NC materials by affecting the deformation process dominated by the GB behaviors.

The research based on molecular dynamics simulations that was conducted by Keblinski et al. indicated the Coble creep (the creep process dominated by GB diffusion) is one of the main creep mechanisms of ultrafine grained materials [32,33]. Therefore, the GB diffusivity, which was proved to be affected by the orientation of GBs, interface atoms diffusion, and the segregation of solute atoms at GBs, is a very important factor affecting the creep of NC materials [34,35,36]. Thus, is it possible to regulate the creep resistance of NC materials through the segregation of solute atoms at GBs? Some hints can be obtained from existing studies. For example, the simulation studies performed by Schafer et al. showed that the segregation of some certain types of solute atoms at GBs can improve the creep resistance of Cu materials significantly, and different sizes of the second-phase atoms have different influences [37]. The experiments based on Mg alloys also indicated that the segregation of solute atoms can improve the creep resistance of materials [38]. However, at present, there is still a lack of systematic research on the effect of segregation on the creep properties and mechanisms of NC materials. More importantly, understanding the creep mechanisms of ultrafine grained metal materials and their alloys at atomic scale is essential for the design of materials with stable mechanical and thermodynamic properties under various working conditions.

To study the creep properties of NC Ni with different grain sizes and concentrations of second-phase atoms at GBs under different external conditions systematically, a series of large-scale molecular dynamics simulations are carried out in this paper. The corresponding creep mechanisms are also revealed.

## 2. Simulation Methods and Parameters

**Figure 1:**
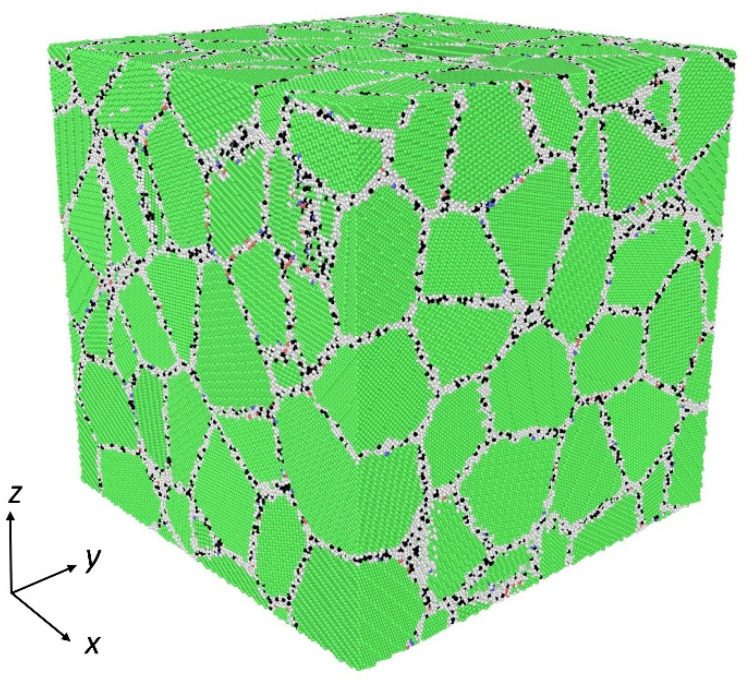
Atomic configuration of segregated polycrystalline Ni-Mo model with *d* = 7.9 nm.

All the three-dimensional polycrystalline models used in this paper are constructed by the ATOMSK software (ATOMSK-Beta 0.10.6, Pierre Hirel, Villeneuve d’Ascq, France) based on the Voronoi method [39]. Three types of models are constructed: the first one is the NC Ni sample without impurity atoms, the second type is the NC Ni sample with a certain proportion of Mo atoms randomly distributed in grains, and the third type is the NC Ni sample with a certain proportion of Mo atoms segregated at GBs, examining the effect of segregation on the creep behaviors of NC materials. The detailed simulation parameters are shown in Table 1. The dimensions of all the models are 35 nm × 35 nm × 35nm, containing about 4 million atoms. The average grain sizes range from 7.9 nm to 19.8 nm. Figure 1 gives the atomic configuration of the 3D polycrystalline Ni sample with the grain size of 7.9 nm, in which green and white atoms represent Ni atoms located in the grain interior and GBs, respectively, and black atoms represent the Mo atoms segregated at GBs. The influences of the addition and segregation of Mo atoms on the creep behaviors of NC Ni are studied through the cross-comparison of three types of models. The effects of applied stress, temperature, and grain size on the creep deformation are further investigated using the NC Ni sample with Mo atoms segregated at GBs.

All simulations in this work are performed using the LAMMPS package (LAMMPS-22 August 2018, Sandia National Laboratories, Albuquerque, NM, USA) [40]. The velocity-Verlet integration algorithm is used with the time step being set to 2 fs. The interaction between Ni and Mo atoms is described by the embedded atom potential parameterized by Zhou et al. [41], which can accurately predict some basic properties of materials, such as the lattice parameter, elastic constants, bulk modulus, vacancy formation energy, and so on. Besides, the GB energy of Ni calculated using this potential is in the range of the mean value of GB energy for the NC metals. Periodic boundary conditions are used in all three directions during the simulation. In order to accelerate the process of creep deformation with practical simulation time, a high temperature of 1100 K is adopted in all the simulations except for those used to study the effect of temperature.

During the simulation, each sample is first relaxed by the conjugated gradient method to get the equilibrium configuration at 0 K. Then, by using the Nosé-Hoover method [42], the sample is relaxed under an isothermal-isobaric ensemble for 200 ps to obtain the equilibrium configuration at the given temperature. Starting from the equilibrium, a constant stress is applied in the *y* direction for 200 ps to simulate creep load. The pressures in *x* and *z* directions are kept zero during the simulation. The microstructures are identified by the common neighbor analysis method [43], the face-centered-cubic, hexagonal-close-packed and non-structured atoms are colored green, red, and white, respectively.

## 3. Results and Discussion

### 3.1. Effect of Segregation on Creep Behavior

**Figure 2:**
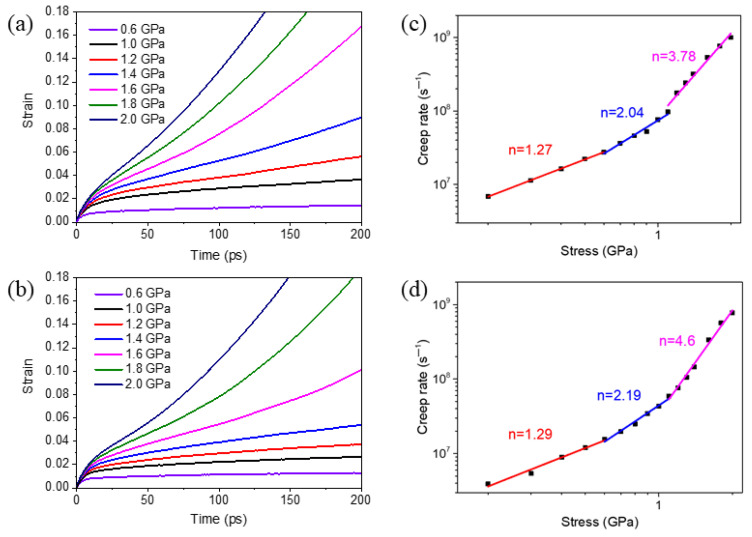
(**a**,**b**) Creep curves of the NC Ni and the segregated Ni-Mo (3 at.%) samples with *d* = 10.9 nm at 1100 K under various applied stresses, respectively; (**c**,**d**) Dual logarithmic plots of the creep strain rate versus stress of NC Ni sample and segregated Ni-Mo (3 at.%) samples with *d* = 10.9 nm at 1100 K, respectively.

To investigate the effect of the segregation of Mo atoms on creep behaviors of NC Ni, creep simulations of the pure Ni samples and the Ni samples with Mo atoms segregated at GBs (referred as segregated Ni-Mo sample thereinafter) are carried out. Figure 2a,b exhibits the evolution of creep strain with respect to time in the 10.9 nm grained Ni and segregated Ni-Mo (3 at.%) samples under different constant stresses at 1100 K. According to the variation of the strain rate on each curve, a typical creep curve under a low stress can be divided into two stages: the initial creep stage and the steady creep stage. During the initial creep stage, the creep strain increases rapidly, the creep strain rate is very large, but gradually decreases with the increase of time. The creep strain rate in the initial stage reflects the instantaneous change of creep strain with time. During the steady creep stage, the strain increases almost linearly with time, implying that the creep rate is almost constant. Under high stresses, the third stage, namely the accelerated creep stage, could be observed, as shown in the curves corresponding to the stress over 1.4 GPa in Figure 2a and over 1.6 GPa in Figure 2b. In this stage, the creep strain rate increases gradually with time until the material breaks. It can also be found that in a certain time range, the critical stress for the third creep stage of the segregated sample is higher than that of the pure Ni sample, which indicates that the segregation of the second-phase atoms at GBs can improve the ability of NC materials to resist creep deformation.

By fitting the slope of creep curves in the steady state stage, the creep rates under different stresses are obtained. Then, the creep rates versus different applied stresses of pure Ni samples and segregated Ni-Mo samples are plotted in Figure 2c,d, respectively. Figure 2c,d shows that the creep rates of both the pure Ni samples and segregated Ni-Mo samples increase significantly with the increase of applied stress. According to the Bird-Dorn-Mukherjee equation, i.e., Equation (1), the stress exponent *n* can be obtained by fitting the slope of the dual logarithmic graph of creep rate and stress, which essentially reflects the strain rate sensitivity of the materials. Here, it can be seen from Figure 2c,d that in the low stress range, the values of *n* for both the pure Ni sample and segregated Ni-Mo sample are greater than 1 and less than 2, indicating that atoms diffusion dominates the creep deformation process within this stress range, but there is also a GB slip phenomenon during the deformation. In practical terms, GB diffusion and GB slip are generally coupled because the diffusion of the atoms at GBs will lead to the change of grain morphology, so the deformation compatibility at the GBs should be maintained by sliding between grains [19]. In the medium stress state, the values of *n* for pure Ni and segregated Ni-Mo samples are about 2, which implies that the GB slip is the main deformation mechanism within this stress range. In the high stress state, for pure Ni samples, the stress exponent approaches 4, suggesting that both the GB sliding and dislocation activities exist in the creep deformation process, but the dominated mechanism is the dislocation activities; for segregated Ni-Mo samples, the stress exponent is greater than 4, implying that the creep is governed by dislocation activities.

**Figure 3:**
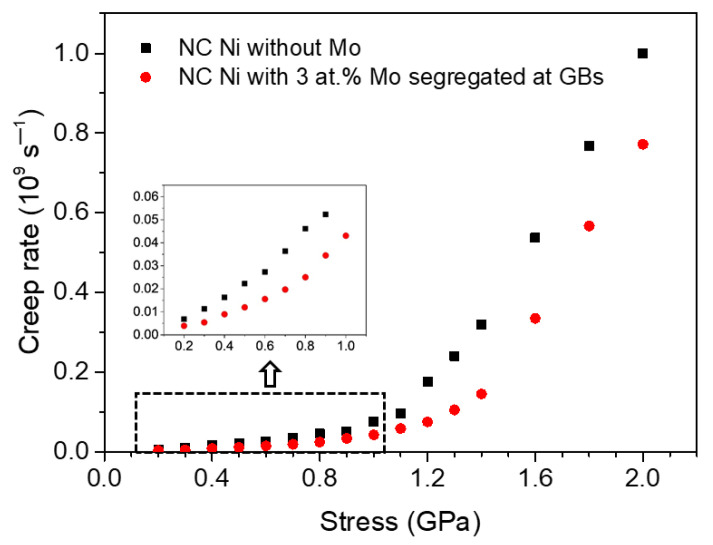
The comparison of the creep strain rate of two groups of NC Ni samples with *d* = 10.9 nm at 1100 K under different applied stresses.

By comparing Figure 2c,d, it can be seen that although the stress exponent values of the segregated Ni-Mo sample are larger than those of pure Ni sample, the former is lower than the latter for a given stress. To further see the difference of creep rate between the two NC Ni samples more clearly, we put the creep rate of the two samples under different stress into the same figure, as shown in Figure 3. It can be found that the creep rate of polycrystalline Ni decreases significantly when there are Mo atoms segregated at the GBs, and the strengthening effect becomes more obvious with the increase of stress within a certain stress range.

**Figure 4:**
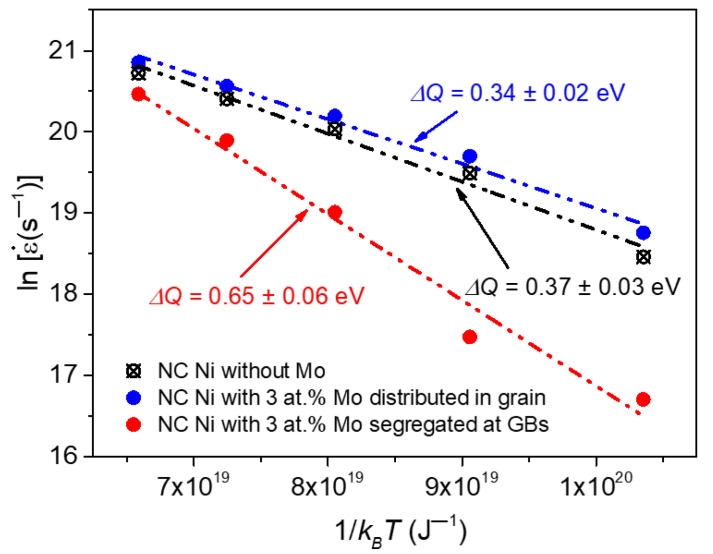
Arrhenius plot of the creep rate against the reciprocal temperature of NC Ni with *d* = 10.9 nm under an applied stress of 2 GPa.

To eliminate the influence of the addition of Mo atoms on the creep resistance of NC Ni, and further prove that the increase of creep resistance of NC Ni is due to the segregation effect at the GBs, we conduct a creep simulation of a group of the NC Ni samples with Mo atoms randomly distributed in the grains. The results show that the creep rate of the NC Ni sample with Mo atoms distributed in the grains is slightly higher than that of the pure Ni sample. Through linearly fitting the relationship between the creep rate and reciprocal temperature of these three sets of NC Ni models, the activation energies during the creep are obtained, and the results are shown in Figure 4. The results reveal that the activation energy of the segregated Ni-Mo sample is about 0.65 eV, which is significantly higher than that of the NC Ni sample without Mo atoms. However, the activation energy of the Ni sample with the Mo atoms distributed in the grains is about 0.34 eV, which is slightly lower than that of pure Ni sample. It should be noted that all the activation energies derived from molecular dynamics simulations are in the same order of magnitude as those observed in experiments [9]. The results show that the addition of Mo atoms randomly distributed in the grains reduces the activation energy of NC Ni, making creep easier to occur. This may be due to the lattice distortion caused by Mo atoms distributed in the grains, which increases the free energy of the material, and thereby reduces the external activation energy required for creep. The results also reveal that the segregation of Mo atoms at the GBs can visibly improve the activation energy of the material, which makes the creep difficult to occur. This also proves that the remarkable improvement of the creep resistance of NC Ni is due to the segregation effect of Mo atoms at the GBs.

Based on the analysis above, it can be seen that the NC Ni materials have better creep resistance when there are Mo atoms segregated at the GBs, which is consistent with some previous simulation and experimental results [37,38]. In the next section, the potential creep mechanism of NC materials under different applied stresses and the strengthening mechanism of segregation are discussed in detail.

### 3.2. Creep Mechanism Analysis

**Figure 5:**
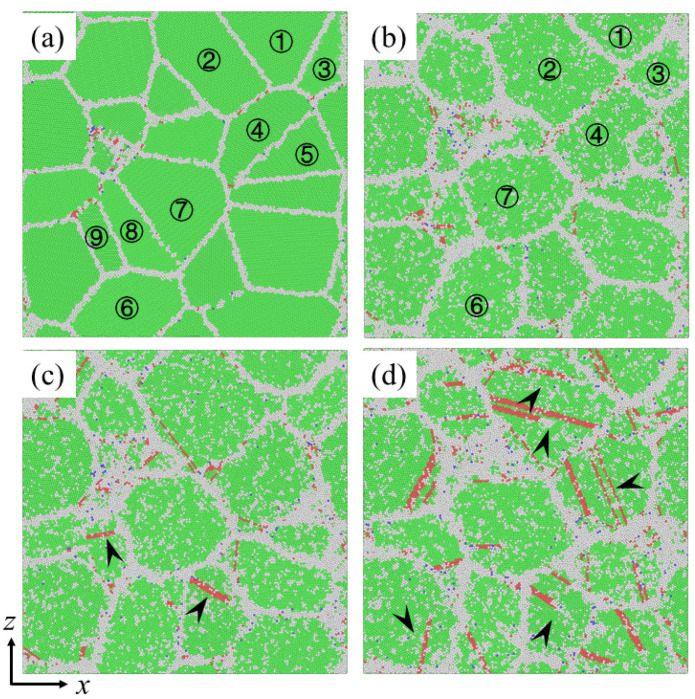
Snapshots of a pure Ni sample with *d* = 10.9 nm at 1100 K (**a**) before creep; (**b**–**d**) after creep for 200 ps under an applied stress of 0.6 GPa, 1.0 GPa and 1.6 Gpa, respectively.

To better understand the inherent mechanisms of the effect of segregation at GBs on creep behaviors of NC materials, the microstructure evolution of NC pure Ni and segregated Ni-Mo samples under different stresses are investigated. Figure 5a exhibits the initial atomic configuration of the 10.9 nm grained pure Ni sample, and the deformed configurations of this sample after creep for 200 ps at the stress of 0.6 GPa, 1.0 GPa, and 1.6 GPa are shown in Figure 5b–d, respectively. In Figure 5b, the grain morphology changes obviously after creep at the stress of 0.6 GPa, compared with the initial atomic configuration before creep in Figure 5a. The grains with obvious morphological changes are marked by numbers in Figure 5a,b: the sizes of grains 2 and 3 increase obviously, while the size of grain 1 between them decreases; grain 4 gobbles up grain 5 gradually; grains 6 and 7 grow while grains 8 and 9 decrease in size and merge into one grain. These phenomena indicate that GB diffusion and GB slip occurred during the creep deformation. Besides, there is no dislocation found in the atomic configuration, implying that the dominant creep mechanisms under low stress states are the GB diffusion and GB slip, and this is consistent with the prediction of Figure 2c based on Equation (1). When the applied stress is 1.0 GPa, in addition to the obvious change of grain morphology, a small amount of dislocations are also observed in the atomic configuration, as indicated by the white arrows in Figure 5c, which illustrates that the prevalent mechanisms of the creep deformation are still GB behaviors, accompanied with a few dislocation activities. Figure 5d is the configuration after creep under the stress of 1.6 GPa, it is obvious to find that extensive partial dislocations nucleate at the GBs, and then pass through the grain interior, leaving behind stacking faults, as indicated by the white arrows. This indicates that the dislocation activities play a more important role during the creep deformation under high stress, which is also in line with the prediction of Figure 2c based on Equation (1).

**Figure 6:**
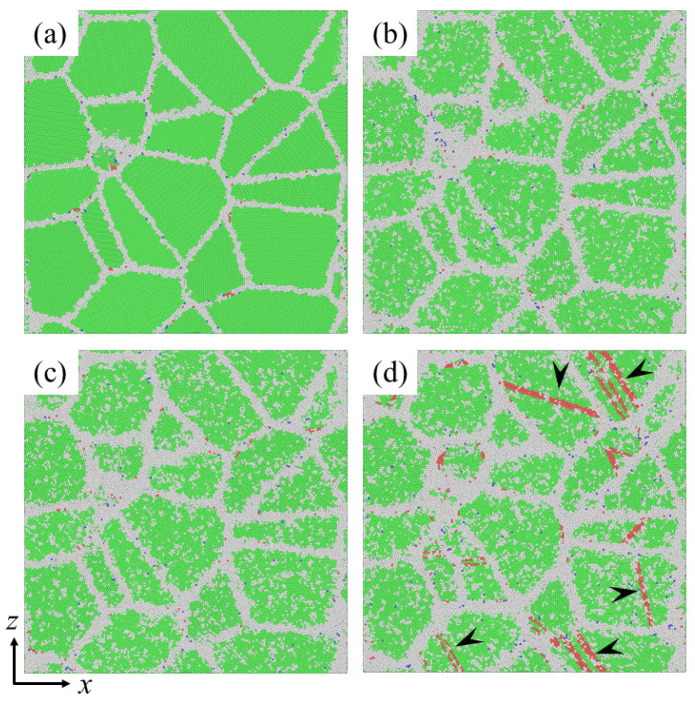
Snapshots of segregated Ni-Mo (3 at.%) samples with *d* = 10.9 nm at 1100 K (**a**) before creep; (**b**–**d**) after creep for 200 ps under an applied stress of 0.6 GPa, 1.0 GPa and 1.6 GPa, respectively.

Figure 6a–d shows the atomic configurations of segregated Ni-Mo (3 at.%) samples before creep and after creep for 200 ps under various applied stresses, respectively. Comparing Figure 6b,c with the initial atomic configuration in Figure 6a, it is found that the grain morphologies do not change obviously and there are almost no dislocation activities. Combined with the creep mechanisms predicted above according to Figure 2d, this implies that in the low and medium stress area, the creep deformation of segregated Ni-Mo sample is very small. Therefore, it can be found that the segregation of Mo atoms at GBs can effectively inhibit the creep process mediated by GB behaviors, thus improving the creep resistance of NC materials. When the stress is 1.6 GPa, a large number of stacking faults are observed in the grain interior, as indicated by the white arrows in Figure 6d. This means that the creep deformation is governed by dislocation activities when the applied stress is in the high stress regime, which is consistent with previous analysis [9,10].

### 3.3. Influences of Grain Size and Temperature

**Figure 7:**
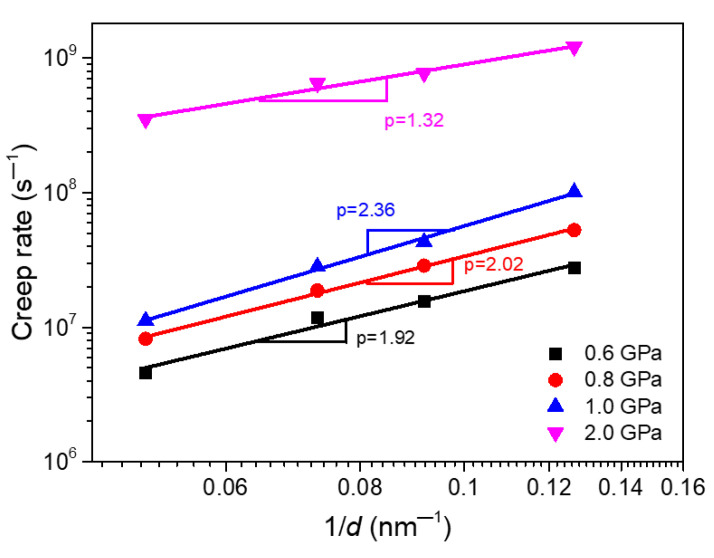
The relationship between the creep strain rate and the reciprocal of grain size *d* of the segregated Ni-Mo (3 at.%) sample at 1100 K under different applied stresses.

Generally speaking, as surface defects, the GBs in NC materials are in thermodynamically non-equilibrium state, and the creep is essentially a thermal activation process, so the GB and temperature both have important influence on the creep properties of materials. Therefore, this section focuses on the effects of grain size and temperature on the creep behaviors of segregated Ni-Mo samples. According to Equation (1), we can know that the creep strain rate will decrease with the increase of grain size for a fixed temperature and applied stress. The effect of the grain size on creep behaviors of the material is mainly characterized by the grain size exponent *p*. Figure 7 gives the relationship between the creep strain rate and the reciprocal of grain size of segregated Ni-Mo (3 at.%) sample under different applied stresses at 1100k. The double logarithmic plot in Figure 7 implies that the creep rate always increases with the decrease of grain size for all stress levels, and in a power-law relationship. From the figure, we can also see that the grain size exponents are about 1.92–2.36 for the applied stress within the low and medium stress range in Figure 2d. When the stress is in the range of 0.6–0.8 GPa, the values of stress exponent *p* are close to 2, which just corresponds to the Nabarro-Herring creep. When the stress continues to increase to 1.0 GPa, the stress exponent *p* is greater than 2 and less than 3, but closer to 2, which indicates that both the Nabarro-Herring and Coble diffusion or GB slip exist in the creep deformation process, but the dominant creep deformation mechanism is still the Nabarro-Herring diffusion. It can be seen that the mechanisms of lattice diffusion, GB diffusion and GB slip coexist under the low and medium stress state, but the segregation of Mo atoms at the GBs dramatically impedes the creep process mediated by GB behaviors. In addition, it should be noted that when the stress is 2.0 Gpa, within the high stress range in Figure 2d, the grain size exponent is 1.32, which means that the dislocation activities dominate the creep process.

**Figure 8:**
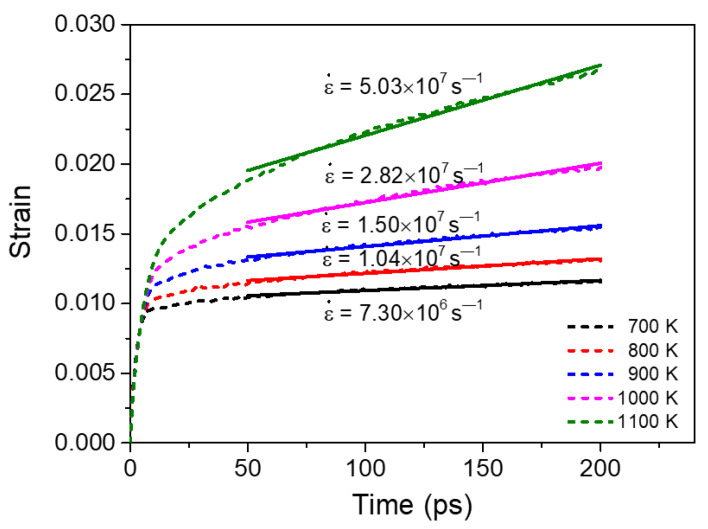
Creep curves of the segregated Ni-Mo (3 at.%) sample with *d* = 10.9 nm at different temperatures under an applied stress of 1.0 GPa.

Figure 8 exhibits the creep curves of segregated Ni-Mo (3 at.%) sample with grain size of 10.9 nm under an external stress of 1.0 GPa at different temperatures. It can be seen that for the segregated NC material, the creep rate increases with the increase of temperature, which is similar to that of pure NC materials [19,20,44]. This is because all creep mechanisms are essentially thermal activation processes.

### 3.4. Effect of Segregated Atoms Concentration at GBs

**Figure 9:**
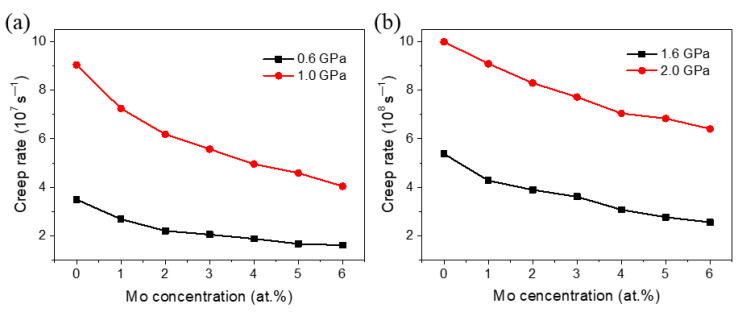
Variation in creep strain rate with the Mo concentration of segregated Ni-Mo samples with *d* = 10.9 nm under different stresses: (**a**) 0.6 GPa and 1.0 GPa, and (**b**) 1.6 GPa and 2.0 GPa.

According to our previous quantitative research on the influence of segregation on GB energy of Ni [26], it can be known that, within a certain range, the GB energy decreases linearly with the increase of the concentration of Mo atoms at GBs, and the concentration has a significant effect on the plastic deformation process dominated by GB behaviors of NC Ni. The GB behaviors are also the important creep mechanisms of NC materials, so the concentration of Mo atoms segregated at the GBs also has an important influence on the creep behavior of NC Ni. To understand this influence more clearly, this section mainly investigates the creep behavior of polycrystalline Ni with different concentrations of Mo atoms segregated at GBs. Figure 9 shows the variation of creep strain rate with the concentration of Mo atoms segregated at GBs for the sample with an average grain size of 10.9 nm under different stresses. It can be seen that whether in the low stress regime or the medium and high stress regimes, as the Mo atoms concentration increases from 0 to 6 at.%, the creep strain rate of the material gradually decreases. This demonstrates that the creep resistance of NC materials can be optimized by adjusting the concentration of the second-phase atoms segregated at GBs. This may be useful for the design and fabrication of NC materials with excellent creep resistance.

In addition, it can also be found from Figure 9 that when the stress is between 1.0 GPa and 2.0 GPa, the influence of the concentration of Mo atoms on the creep strain rate is stronger than that when the applied stress is 0.6 GPa, which is due to the different creep mechanisms of materials under different stress states. From our previous work, we can know that the segregation of solute atoms at GBs can significantly improve the stability and reduce the mobility of GBs [26], which implies that the segregation of second-phase atoms at GBs has a greater influence on the creep process dominated by GB behaviors, such as when the stress is 1.0 GPa. From the analysis of grain size exponent above, it can be known that when the stress is 0.6 GPa, the deformation corresponds to the Nabarro-Herring creep, that is, the creep process is dominated by the lattice diffusion. Therefore, the segregation of Mo atoms at GBs has little effect on the creep rate, and with the increase of the concentration of Mo atoms, the creep rate quickly reaches a steady state and almost no longer decreases. Moreover, it is worth noting that the results in Figure 9 show that in the high stress region, i.e., 1.6 GPa and 2.0 GPa, the segregation of Mo atoms at GBs also has a significant effect on the creep process, while the analyses of stress and grain size exponents both indicate that the dominant mechanism of creep deformation is dislocation activities within this stress regime. This means that the segregation also has striking effect on creep deformation dominated by dislocation activities.

**Figure 10:**
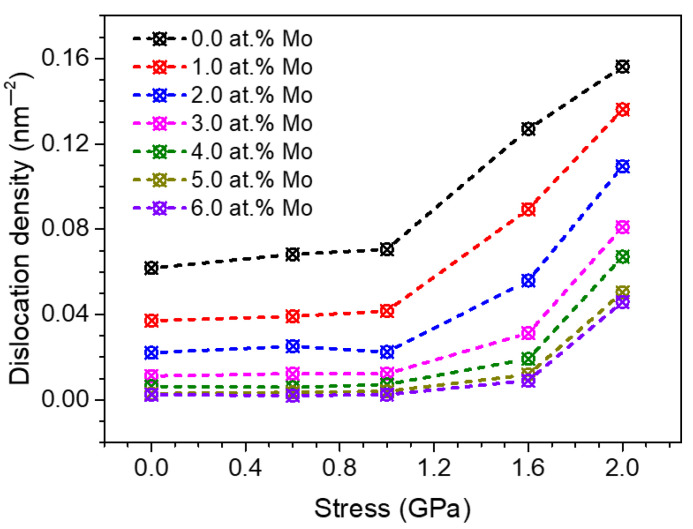
Relationship between the dislocation density and the applied stress in segregated Ni-Mo samples with different Mo concentrations after creep at 1100 K for 200 ps, the situation of stress at 0 GPa represents the dislocation density of the equilibrium configuration at 1100 K.

To explore the intrinsic mechanism of the influence of segregation on creep deformation dominated by dislocation activities, the dislocation densities of the 10.9 nm grained segregated Ni-Mo samples with different Mo concentrations after creep for 200 ps at 1100 K are shown in Figure 10. The corresponding data points at 0 GPa represent the dislocation density of equilibrium configuration before loading. It can be clearly seen from Figure 10 that when the stress is 0.6 GPa or 1.0 GPa, for all segregated Ni-Mo samples, the dislocation densities after creep 200 ps are basically the same as that in the corresponding equilibrium configurations. This also proves that there is almost no dislocation nucleation during the creep under low and medium stress states, and the creep deformation is dominated by the diffusion and GB sliding, in accordance with the previous analysis. When the applied stress increases to 1.6 GPa or 2.0 GPa, it can be found that the dislocation densities of all samples increase significantly compared with the equilibrium configurations. This trend also proves that the main creep mechanism under a high stress state is the dislocation activity. It should be noted that the dislocation densities of segregated samples with different Mo concentrations are much lower than that of the pure Ni sample, and the higher the proportion of Mo atoms, the lower the dislocation density in the segregated sample, which indicates that the segregation of Mo atoms at GBs inhibits the nucleation and proliferation of dislocation in NC Ni during the creep. When the proportion of Mo atoms is higher than 4 at.%, the dislocation density in the material under the applied stress of 1.6 GPa is even reduced to the same level of equilibrium configuration.

**Figure 11:**
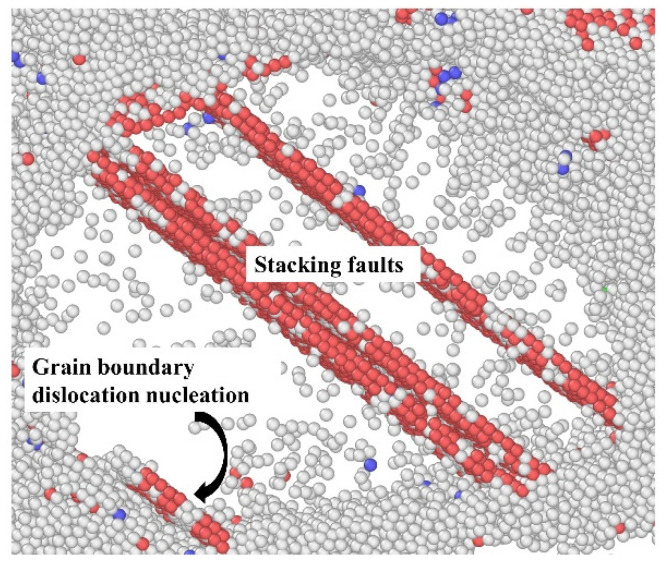
Dislocation nucleation from GBs and the left stacking faults during creep deformation under an applied stress of 1.6 GPa at 1100 K. The snapshot is from the segregated sample with 6 at.% Mo atoms and *d* = 10.9 nm, in which the perfect fcc atoms are not shown for clarity.

To show more evidences about the proposed dislocation activity as the dominant mechanism under high stress, we picked one grain among the 10.9 nm grained segregated Ni-Mo (6 at.%) sample during the steady state creep at 1.6 GPa, as shown in Figure 11. It can be obviously found that due to the limitation of grain size, the dominant creep mechanism is the dislocation nucleation from GBs rather than the collective dislocation dynamics inside grains, which has been confirmed by the previous studies on the deformation mechanism of NC materials [45]. By comparing the dislocation density of all samples at equilibrium state in Figure 10, it can be found that the dislocation density decreases with the increase of the concentration of Mo atoms, which implies that the segregation of Mo atoms at GBs destroys the dislocation structure at GBs. However, the dislocation structures at the GBs are the sources of dislocation nucleation during the creep, so the segregation of Mo atoms at GBs can also significantly reduce the creep rate of materials under high stress.

## 4. Conclusions

A train of large-scale molecular dynamics simulations are carried out to investigate the influence of the segregation of Mo atoms on the high-temperature creep behavior of NC Ni. The effects of the applied stress, grain size, temperature, and concentration of Mo atoms on the creep behaviors and mechanisms are systematically studied. The main conclusions are summarized as follows:

(1) The segregated Ni-Mo sample corresponds to a higher activation energy than the pure Ni sample, which makes the creep of the NC Ni more difficult to occur and thus enhances the creep resistance of the material;
(2) For both the pure Ni sample and segregated Ni-Mo sample, the creep mechanisms are the diffusion, GB slip, and dislocation activity in the low, medium, and high stress regimes, respectively;
(3) The segregation of Mo atoms at GBs has little effect on the creep processes dominated by the lattice diffusion, but significantly slows down the creep processes dominated by the GB behavior and dislocation activity.

## Figures and Tables

**Table 1:** Parameters in the creep simulation of NC Ni-Mo alloy.

Group	σ (GPa)	d (nm)	T (K)	Mo (at.%)
1	0.2, 0.3, 0.4, 0.5, 0.6, 0.7, 0.8, 0.9, 1.0, 1.1, 1.2, 1.3, 1.4, 1.6, 1.8, 2.0	10.9	1100	0.0, 3.0
2	0.6, 0.8, 1.0, 2.0	7.9, 10.9, 13.7, 19.8	1100	3.0
3	1.0	10.9	700, 800, 900, 1000, 1100	3.0
4	0.6, 1.0, 1.6, 2.0	10.9	1100	0.0, 1.0, 2.0, 3.0, 4.0, 5.0, 6.0

## Data Availability

The raw/processed data required to reproduce these findings cannot be shared at this time as the data also forms part of an ongoing study.
